# A Bioinformatic Pipeline Places STAT5A as a miR-650 Target in Poorly Differentiated Aggressive Breast Cancer

**DOI:** 10.3390/ijms21207720

**Published:** 2020-10-19

**Authors:** Eric López-Huerta, Ezequiel M. Fuentes-Pananá

**Affiliations:** 1 M. Sc. Program in Biochemical Science, National Autonomous University of Mexico, 04510 Mexico City, Mexico; ericko1195@hotmail.com; 2 Research Unit in Virology and Cancer, Children’s Hospital of Mexico Federico Gómez, 06720 Mexico City, Mexico

**Keywords:** miR-650, amplification, STAT5A, target, breast cancer, bioinformatic

## Abstract

Breast cancer (BRCA) is a leading cause of mortality among women. Tumors often acquire aggressive features through genomic aberrations affecting cellular programs, e.g., the epithelial to mesenchymal transition (EMT). EMT facilitates metastasis leading to poor prognosis. We previously observed a correlation between an amplification of miR-650 (Amp-650) and EMT features in BRCA samples isolated from Mexican patients. In this study, we explored the cBioportal database aiming to extend that observation and better understand the importance of Amp-650 for BRCA aggressiveness. We found that Amp-650 is more frequent in aggressive molecular subtypes of BRCA, as well as in high grade poorly differentiated tumors, which we confirmed in an external miRNA expression database. We performed differential expression analysis on samples harboring Amp-650, taking advantage of gene target prediction tools and tumor suppressor gene databases to mine several hundreds of differentially underexpressed genes. We observed STAT5A as a likely putative target gene for miR-650 in aggressive poorly differentiated BRCA. Samples with both Amp-650 and low expression of STAT5A had less overall survival than samples with either or none of the alterations. No target gene has been described for miR-650 in BRCA, thus, this bioinformatic study provides valuable information that should be corroborated experimentally.

## 1. Introduction

Breast cancer (BRCA) is the most common cancer and one of the leading causes of mortality in women worldwide [1]. Tumors become aggressive upon acquiring the ability to detach from the epithelial cell sheet, invade, enter the blood stream, and colonize secondary organs, all of these while evading apoptotic mechanisms. This process is called metastasis and it is importantly controlled by a set of molecules that induce the epithelial to mesenchymal transition (EMT) [2]. EMT is a process in which adherent epithelial cells are converted into mesenchymal cells with the capacity to migrate and invade [3]. It is also proposed that EMT facilitates tumor stemness, forming poorly differentiated cancer cells with self-renewal capacity [4]. Cancers with EMT and stemness features, such as heightened motility and invasiveness, are more aggressive and lead to poor prognosis.

BRCA is classified into molecular subtypes based on the expression of estrogen, progesterone, or human epidermal growth factor 2 (HER2) receptors, as luminal A, luminal B, HER2-enriched, and triple negative (subdivided into basal-like and claudin-low). This classification gives insight into prognosis, being the triple negative and HER2-enriched the ones with the worst outcome [5]. Furthermore, it has been observed that molecular subtypes resemble different states of normal mammary development. Basal, claudin-low, and HER2-enriched resemble early progenitors, sharing high degree of mesenchymal signatures (that is EMT features), while luminal A and luminal B are the most differentiated with a heightened epithelial signature [6,7]. Another classification useful to describe differentiation states of BRCA cells, is the Neoplasm Histologic Grade (NHG), which ranges from grade 1 to grade 3 indicating a gradient that goes from well differentiated to poorly differentiated tumor cells, also with the latter exhibiting the worst prognosis [8]. The Nottingham Prognostic Index (NPI) [9] and TNM staging are also indirect guides of the progression and grade of invasion of cancer cells to adjacent tissues [10]. TNM standing for Tumor size, number of infiltrated lymph Nodes, and the presence of Metastasis.

MicroRNAs (miRNAs) are small noncoding RNAs that negatively regulate gene expression by interfering with mRNA translation. It has been widely reported that miRNAs can be overexpressed in cancer as a consequence of chromosomal translocations and amplifications, or epigenetic regulation. These so called oncomirnas can favor the development of aggressive features in cancer by targeting tumor suppressor genes (TSG) [11]. Identification of the oncomirnas putative target TSG is an area of active research in cancer, usually accomplished by looking into complementary 6–8 nucleotide sequences within the mRNA 3′-UTR region, known as the seed region.

We previously reported that BRCA samples from Mexican patients had in common an amplification in band 22q11.2 in the region that encodes miR-650 (Amp-650), and this amplification correlated with expression of EMT markers and greater invasive capacity of BRCA cell lines originated from the same patients [12]. It has been reported that miR-650 works as an oncomirna in hepatocellular carcinoma, colorectal cancer, prostate cancer, and gastric cancer, targeting ING4, NDRG2, and SCARA3 (also known as CSR1) TSG [13,14,15,16]. However, no target gene of miR-650 has been reported in BRCA yet. We therefore aimed to extend our initial observation and corroborate whether the association between Amp-650 and BRCA with EMT features was certain in a large cohort of patients, the cBioportal database. Furthermore, using miRNAs target prediction bioinformatic tools, we mined several hundreds of differentially expressed genes between samples with and without Amp-650, to find differentially underexpressed (DU-Exp) genes that were most likely bona fide targets of miR-650. We found that 7.1% of the BRCA samples in the cBioportal database harbor Amp-650 and that this genetic abnormality correlates with downregulation of the miR-650 putative target TSG STAT5A in poorly differentiated aggressive BRCA subtypes.

## 2. Results

### 2.1. cBioportal Analysis

We explored the cBioportal database and gathered four different studies that contained suitable information to address questions about the frequency of Amp-650 in BRCA patients, and about its possible association with cancer aggressive features, such as EMT and state of differentiation. Each study provided different information, as shown in Table 1.

### 2.2. Amp-650 Frequency in BRCA Samples

Data for copy number alterations (CNA) regarding the miR-650 chromosomal region were integrated from four different studies to calculate the frequency of Amp-650. To have a better insight into the relevance of miR-650 for BRCA, we also calculated the amplification frequency of reference miRNAs: miR-21, miR-210, and miR-10B, previously reported to be upregulated in BRCA (Table 2). We found that Amp-650 is present in 7.1% of the samples, a similar frequency of two of the reference miRNAs, except for miR-21 that was amplified in 30.6% of samples. Thus, Amp-650 is well represented in a large cohort of BRCA patients. As contrasting data, we observed that the opposite alteration, a deletion of the same region (22q11.2), was only present in 0.1% of the samples.

### 2.3. BRCA Subtypes with Enriched Amp-650

**Figure 1:**
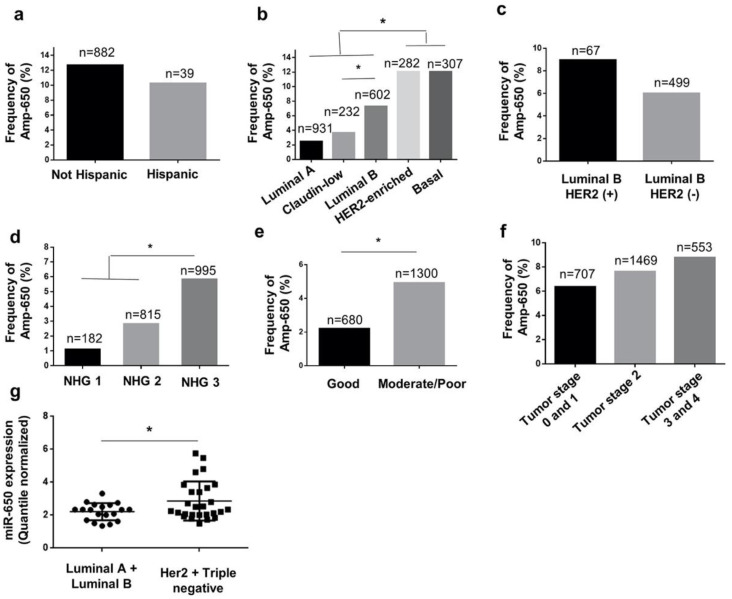
Frequency of Amp-650 in samples with different clinical classifications. The y axis indicates the amplification frequency of miR-650 in samples classified by (**a**) ethnicity, (**b**) molecular subtype, (**c**) HER2 status of Luminal B samples (only samples having both molecular and HER2 data were included). (**d**) Neoplasm Histologic Grade (NHG), (**e**) Nottingham Prognosis Index (NPI), (**f**) tumor stage, and (**g**) differential expression analysis made with GSE86278 dataset over miR-650. Values above each bar indicate the number of samples in each group. Prognosis categorization into good, moderate, and poor, was made based on the NPI thresholds proposed by Albergaria, A. et al. [9] and poor prognosis samples were combined with moderate prognosis samples to increase the group size: poor (*n* = 199) and moderate (*n* = 1101). Likewise, tumor staging 0 (*n* = 12) with 1 (*n* = 695) and 3 (*n* = 323) with 4 (*n* = 30) were merged due to the small sample size. Significant differences are indicated with an asterisk (Z > 1.645).

Based on our previous findings on Mexican BRCA samples with a common Amp-650 [12], we considered that this alteration may be enriched in Hispanic population, however, there was no statistical difference in Amp-650 frequencies between Hispanic (10.26%) and non-Hispanic populations (12.7%) (Figure 1a). Additionally, since we had observed a correlation between Amp-650 and EMT features, we thought that Amp-650 should be enriched in BRCA subtypes with known poor clinical outcomes and increased mesenchymal markers. For this, we assessed whether amp-650 was more represented in BRCA subtypes with more aggressive features considering existing instruments of BRCA classification. Indeed, this was true for most comparisons. For instance, Amp-650 was found significantly more represented in some of the aggressive molecular subtypes: HER2-enriched (12.77%) and basal (12.38%), but also in luminal B (7.48%), with respect to the nonaggressive subtype luminal A (2.47%), while aggressive claudin-low was the only exception (3.88%) (Figure 1b). Considering that luminal B BRCA is molecularly and clinically more heterogeneous than Luminal A, we subclassified the luminal B group according to the protein expression of HER2. We observed that luminal B-HER2 (+) tumors carried the Amp-650 more frequently (8.96%) than luminal B-HER2 (-) (6.01%) (Figure 1c), still, this difference was not significant. Likewise, an analysis based on the histological grade showed that Amp-650 was significantly more frequent in the poorly differentiated, more aggressive NHG 3 samples (5.83%) than in more differentiated histological grades NHG 2 (2.82%) and NHG 1 (1.1%) (Figure 1d). Our initial observation was somehow also supported by the NPI, in which Amp-650 was found significantly more enriched within a combined set of moderate/poor (4.92%) prognosis, than in BRCA samples with good prognosis (2.21%) (Figure 1e). In order to analyze TNM staging, we homogenized data from three different studies (Table 1) and merged tumor stage subgroups into five main groups to increase the number of samples per group. We observed modest differences in TNM staging, with a tendency of Amp-650 to be more frequent in more advanced stages 3 and 4 (8.78%), followed by stage 2 (7.62%), and early stages 0 and 1 with the lowest frequency (6.36%) of Amp-650 (Figure 1f), however, these frequencies were not statistically different. Additionally, we explored miR-650 expression on 46 samples from Mexican patients with molecular subtype classification data, available in a public dataset (GSE86278) (Figure S1). We found that miR-650 was more expressed in aggressive molecular subtypes, HER 2 and triple negative in comparison with nonaggressive luminal A and luminal B (*p*-value = 7.9 × 10^−4^) (Figure 1g). This was in agreement with our observation in Figure 1b.

### 2.4. Putative Target TSG for miR-650

**Figure 2:**
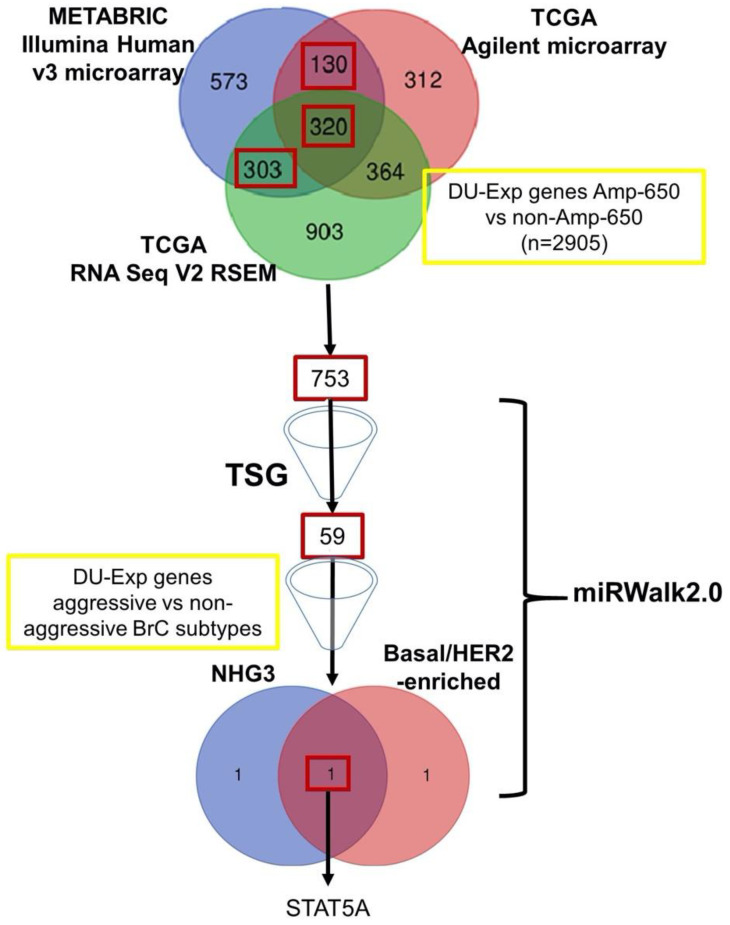
Schematic representation of the workflow followed to mine genes from differential expression analysis of putative targets of miR-650. Red boxes indicate genes that were carried to subsequent analysis. Aggressive breast cancers (BRCA) are a compilation of basal, HER2-enriched, and NHG 3, while nonaggressive are a compilation of luminal subtypes and NHG 2.

The enrichment of Amp-650 in molecular subtypes with poor prognosis, HER2-enriched and basal, and in poorly differentiated BRCA led us to hypothesize that miR-650 may be targeting TSG to promote cancer aggressiveness and progression. This is also in agreement with our previous correlation between Amp-650 and EMT features. To inquire into which TSG may be under Amp-650 transcriptional control, we performed several differential expression analyses comparing different sets of samples with and without Amp-650 (Table 3). The sum of all samples with Amp-650 and without Amp-650 regardless of the BRCA subtype was named as “Total Amp-650”. “Total Amp-650” comparisons were used to begin with gene filtering, as described in the summary of procedures of Figure 2.

We found 2905 DU-Exp genes (*p*-value < 0.01) between samples with and without Amp-650. We placed more stringent filters to reduce this number, first by selecting only those genes that were shared by at least two transcriptomic platforms across METABRIC and TCGA. Genes shared by the two different transcriptomic platforms within the same study (Agilent microarray and RNA Seq V2 RSEM, both from TCGA, *n* = 364) were not taken into account, since we considered that these genes were shared because they came from the same samples. To further reduce the resulting list of 753 DU-Exp genes, we focused on those genes that were predicted to be targets of miR-650 by at least one predicting tool according to miRWalk 2.0, and by focusing on TSG according to the Tumor Suppressor Gene Database. We ended up with 59 genes that fulfilled both conditions. We then addressed if within this group of 59 genes there were TSG that were specifically lost in basal, HER2-enriched, and/or NHG 3 samples but not in Luminal subtypes or in less advanced grade BRCA samples (*p*-value < 0.01). We observed that almost all, except for one, of experimentally documented targets of miR-650 in solid cancers were predicted as targets by seven and six miRWalk tools (Table S1), arguing that six is a reliable threshold for prediction. We found three genes as potential miR-650 targets using the six miRWalk tools as threshold: STAT5A (identified by six predicting tools), THBD (seven), and PER2 (eight) (shown in Table 4). Of them, only STAT5A met the requirement of being a DU-Exp gene in BRCA aggressive subtypes and in poorly differentiated NHG 3. No TSG was predicted as a miR-650 target by all the 12 miRWalk tools (Table S2).

### 2.5. Interrelation of miR-650 Putative Target Genes

**Figure 3:**
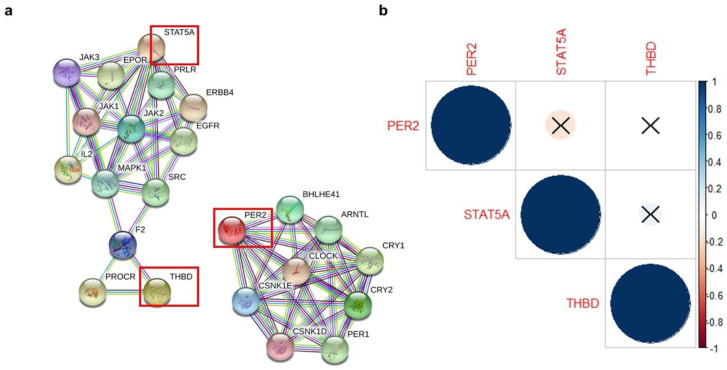
Pathway enrichment analysis of putative target genes and their expression correlation. (**a**) Protein–protein interaction networks. Red squares denote the miR-650 putative target genes used as input in the STRING software. (**b**) Correlation matrix of microarray expression data from METABRIC using samples of basal, HER2-enriched, or NHG 3 subtypes and harboring Amp-650 (*n* = 63). Blue and red colors indicate positive and negative correlations, respectively, the larger the circle the closer the correlation to 1:1, and crossed circles denote a not significant correlation. A similar analysis with TCGA gene expression data also does not support a correlation between these genes (data not shown). Correlation plots generated with corrplot package [19].

We constructed an interaction network to reveal direct and indirect partners of STAT5A, THBD, and PER2 and performed pathway enrichment analyses with the set of genes of the network using STRING (see Section 4.4) (Figure 3a). We observed that these genes did not have direct interactions between them, but there was an indirect connection between STAT5A and THBD through intermediaries. This analysis also found 235 pathways significantly enriched with these genes (FDR < 0.001) (Table S3). In general, we found that STAT5A regulates developmental processes, including mammary gland development (30th position, FDR = 3.2 × 10^−7^), the isolated module of PER2 plays an important role in circadian rhythm (many of the top 10 positions), and THBD retrieves mostly general terms related to cell regulation and signaling. Even though STAT5A and THBD were indirectly connected, their pathways remained largely independent of each other or with minimal intermediaries in common. Furthermore, correlation analysis of their mRNA levels showed that their expression is not coordinated (Figure 3b). These observations further support that these genes participate in different processes independently of each other.

### 2.6. The Prognostic Value of miR-650 Putative Target Genes

**Figure 4:**
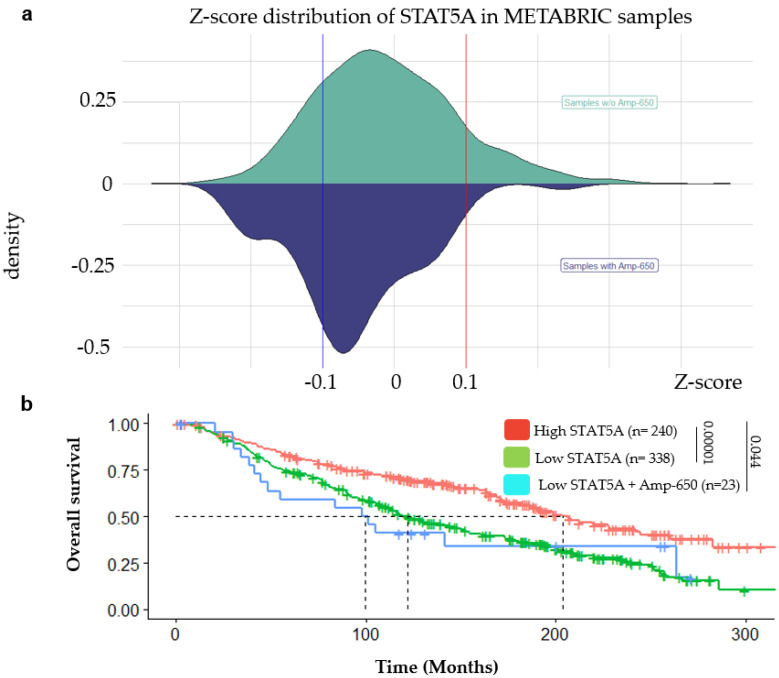
(**a**) Z-score was used to set high expressing samples (above + 1) or low expressing samples (below -1) of STAT5A. (**b**) Prognostic significance of Amp-650 in combination with the expression level of STAT5A. There were only two samples with high STAT5A (STAT5A above + 1) + Amp-650. Dashed lines indicate median OS (Months): High STAT5A = 203.7, Low STAT5A = 122.17, and Low STAT5A + Amp-650 = 99.55. Statistical differences are indicated with a line accompanied by its *p*-value. Samples without amplification (w/o Amp-650). Both plots were constructed with downloaded Z-score distribution of Illumina Human v3 microarray data from METABRIC (*n* = 1980).

Using survival and mRNA expression data from METABRIC, we tested whether downregulation of STAT5A either with or without Amp-650 was influencing the patients’ overall survival (OS). From cBioportal, we downloaded the Z-score distribution of STAT5A expression in samples with and without Amp-650, observing a tendency for lower expression in samples with the amplification (Figure 4a). We used this analysis to establish the Z-scores thresholds for high and low STAT5A expression (see Section 4.5). We then compared low and high expression levels of STAT5A alone and with or without Amp-650. Since we could only find two samples with high STAT5A expression and Amp-650, we could not carry out statistical comparisons with this group, and we ended up with only three groups. We found that patients with low expression of STAT5A have significantly lower OS than those with high expression of STAT5A (*p*-value = 0.00001) (Figure 4b), median OS of 122.17 and 203.7 months, respectively. More importantly, patients in whom low expression of STAT5A and Amp-650 concurred were the ones with the worst survival (99.55 months). We found significant difference between this group and the groups of high STAT5A (*p*-value = 0.044). Using expression data from the TCGA, samples with Amp-650 together with low expression of STAT5A had also the worst OS than any other group but showed no statistical significance (Figure S2a,b). We did not find statistical differences for THBD and PER2, not even in combination with Amp-650, suggesting that these genes are not good markers for the survival of BRCA patients (Figure S3a–f).

## 3. Discussion

We previously observed Amp-650 in BRCA samples derived from Mexican patients with mesenchymal/invasive tumors [12]. Here, we explored public databases to assess whether Amp-650 was a recurrent event in BRCA, and whether we could support an important role specific for aggressive subtypes. We determined that miR-650 has a frequency of amplification as high as that of miR-210 and miR-10B, but four times lower than miR-21, yet, miR-21 is one of the most widely reported oncomirnas in a great variety of human cancers [20,21]. Additionally, despite our previous observation, we did not find Amp-650 more represented in the Hispanic population, with the caveat that the databases only included a reduced number of Hispanic patients (*n* = 39), preventing a robust analysis.

Remarkably, Amp-650 was significantly more represented in basal, HER2-enriched, and NHG 3 tumors. Basal, HER2-enriched, and claudin-low have been reported to resemble poorly differentiated progenitor stages of mammary development [7]. It is tempting to speculate that miR-650 may play a role during those early differentiation stages, although this was not correlative with claudin-low tumors. We observed that Luminal B-HER2 (+) samples harbored the Amp-650 more frequently than Luminal B-HER (-), although this difference was not significant, perhaps because of the small representation of luminal B-HER2 (+) samples (*n* = 67). Luminal B carries significantly worst prognosis than Luminal A, and it represents a highly heterogenous molecular subtype with various degrees of expression of estrogen and progesterone receptors, and proliferation index, besides the variable HER2 status [22,23,24]. Indeed, Luminal B tumors are a therapeutic challenge and combinations of endocrine therapy, traditional chemotherapy, and HER2 inhibition has been proposed to level its clinical response.

No previous direct associations have been reported between miR-650 and both STAT5A and PER2, but miR-650 targets have never been explored in BRCA. Interestingly, PER2 is known to control cell growth of acute myeloid leukemia (AML) and lymphoma cell lines, and PER2 reduced expression was confirmed in AML patients [25]. In addition, genomic alterations in the 22q11.2 region are consistently reported in hematopoietic malignancies [26,27,28]. Concerning THBD, a previous association with miR-650 has been reported in thyroid cancer [29]. In that study, a proteomic analysis showed increased levels of THBD upon miR-650 inhibition. Taken together, these data suggest an important cancer participation of an altered expression of miR-650 through 22q11.2 alterations by targeting different TSG, such as PER2 in leukemias and lymphomas, THBD in thyroid cancer, and both of them plus STAT5 in BRCA. This also supports that miR-650 gene repression may be tissue specific. Although our pipeline of analysis combined METABRIC and TCGA data to find Amp-650 DU-Exp genes, once we had potential TSG targeted by miR-650, all three candidates STAT5A, PER2, and THBD were found in METABRIC data specifically downregulated in basal, HER2-enriched, and NHG 3 BRCA. We did not find any TSG targeted by miR-650 in the TCGA that were predicted by six or more miRWalk tools, perhaps because the number of samples with molecular subtype data in this database were 4-fold less than those harbored in METABRIC.

In addition to CNA, we intended to complement our analysis pipeline with miR-650 expression data. However, high throughput transcriptomic datasets that effectively detected miR-650 together with CNA data were, to the best of our knowledge, nowhere available for BRCA samples. This may be due to technical complications with high throughput screening of the 22q11.2 region. Moreover, several miRNAs, such as miR-21, do not have available transcriptomic data in cBioportal, suggesting that measuring miRNAs with high throughput technics might be challenging. Indeed, miRNAs expression is usually measured with microarrays dedicated exclusively for miRNA detection, and miR-650 expression has only been reported through RT-qPCR [13,14,15,16,29,30]. Still, we were able to analyze external miRNA expression data finding an overall augmented expression of miR-650 in aggressive BRCA subtypes, particularly for triple negative samples (conformed mostly of basal-like). Although some triple negative samples showed low expression of miR-650 (Figure S1), this was expected since it is possible that these samples lack the Amp-650.

When we were addressing potential target genes of miR-650, we had unique observations related to BRCA. On one hand, previously reported miR-650 targets, such as SCARA3, were found with significant *p*-values throughout our pipeline, but this gene was not considered for our analysis because it is not included in the TSG database (Table S4). On the other hand, taking ING4 and NDRG2 as examples, even though these are TSG documented targets of miR-650, we did not find them as DU-Exp in Amp-650 positive samples. Expression of these genes is reported to be reduced in BRCA samples compared with normal breast tissue. It is likely that miR-650 independent mechanisms of downregulation are in place. Indeed, NDRG2 is repressed by promoter methylation [31], and deletion of ING4 has been found in 16% of BRCA samples, furthermore, another seven miRNAs downregulate ING4 [32,33], reducing miR-650 influence on ING4 through competition. Additionally, our approach emphasized TSG specifically lost in aggressive BRCA subtypes with EMT and undifferentiated features.

STAT5A was the most likely candidate to be targeted by miR-650 and this may influence some aggressive features of BRCA, since it was preferentially downregulated in Amp-650 positive basal, HER-enriched, and NHG 3 samples. There are two isoforms of STAT5, STAT5A, and STAT5B that share more than 90% of its amino acid sequence. Both STAT5 proteins form homodimers or heterodimers that activate transcription in response to several growth factors, such as prolactin, EGF (epidermal growth factor), insulin, and ErbB4, regulating differentiation, survival, and proliferation pathways in mammary epithelial cells [34,35,36]. Indeed, STAT5 is critical for the development of two cell lineages, T cells and mammary alveolar cells generated during pregnancy. While STAT5B plays a more prominent role in T cell development, STAT5A is essential for generation of luminal progenitors from stem cells that differentiate into mammary alveolar cells [36,37]. In mammary tissue, STAT5A represents the majority of STAT5 levels [34] and exerts a more prominent effect on normal mammary gland development and in cell motility, as observed in an in vitro model where STAT5A and STAT5B were silenced by siRNAs [38].

**Figure 5:**
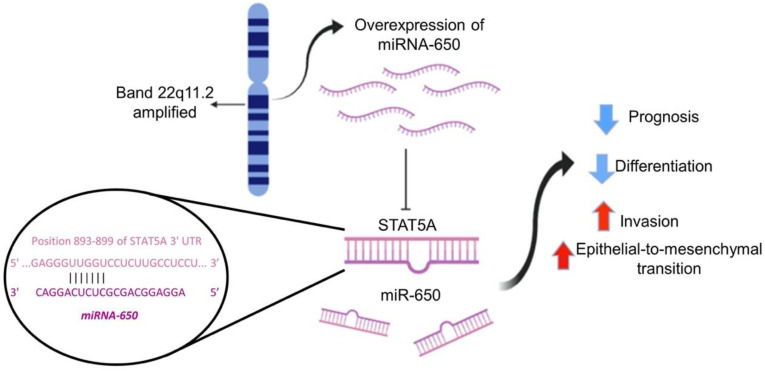
Amp-650 leads to overexpression of miR-650, which causes downregulation of STAT5A. Low expression of STAT5A contributes to the formation of BRCA aggressive subtypes due to facilitating EMT-related features. Closeup circle shows miR-650 seed region and STAT5A sequence complementarity according to TargetScan (http://www.targetscan.org/vert_72/). The main drawing was made with the free version of BioRender (https://biorender.com/).

Although low STAT5A alone denotes poor survival (median OS = 122.17 months), we observed that low levels of STAT5A expression together with Amp-650 mark BRCA patients with worst survival (media OS = 99.55 months). Remarkably, only in two samples concurred high STAT5A expression and Amp-650. Low levels of STAT5 have been previously associated with poor prognosis [39] and with high grade cancers [40], on the contrary, high STAT5 expression has been associated with well differentiated adenocarcinomas and with better prognosis [41,42,43]. It is also worth mentioning that experiments on different BRCA cell lines showed that overexpression of STAT5 induced mesenchymal-to-epithelial transition, the reverse of EMT, and this event correlated with reduced cell invasion [44]. PER2 and THBD did not show reduced OS in any condition with or w/o Amp-650, perhaps because their participation in BRCA might be independent of the development of aggressive features such as EMT, stemness, and invasion. This is also supported by our interacting network and correlation analyses that argue that these genes participate in independent pathways, and although some STAT5A and THBD neighbors are shared in some pathways, STAT5A and THBD remain mostly ontologically separated. Furthermore, the expression of the three genes is not coordinately regulated in BRCA. Figure 5 shows our current working model to explain the miR-650 targeting of STAT5A and the influence that miRNA-650 may exert over BRCA aggressive features. Still, the observations provided in this study with bioinformatic tools should be validated experimentally.

## 4. Materials and Methods

### 4.1. Description of Database Elements of Analysis and Data Treatment

CNA, mRNA expression levels, and patient clinical data, such as the molecular subtype (basal, claudin-low, HER2-enriched, luminal B, and luminal A), ethnicity (Hispanic and not Hispanic), TNM tumor stage, NPI, NHG, HER2 status determined by immunohistochemistry, and OS (months and status) were obtained from cBioportal for Cancer Genomics [45] (https://www.cbioportal.org/) (July 2018), a website that contains genomic and clinical data from different BRCA studies. For mRNA expression data we used METABRIC and TCGA (Firehose legacy) databases that include gene expression from different platforms (Table 1). Table S5 depicts all the features per sample, addressed in the database.

cBioportal classifies CNA data as Gain and Amplification, which stand for low- and high- number of copies of focal amplifications, respectively. For this analysis, regardless of the number of copies that each one represents, we combined both Gain and Amplification when denoting the samples in which the 22q11.2 region was amplified (Amp-650) in order to have a single value for amplification. cBioportal also provides information about homozygous deletion (designated as Deep deletion). We calculated frequencies of samples with both Amp-650 and Deep deletion of the 22q11.2 region.

GSE86278 expression dataset [46] is publicly available and a more detailed description of this dataset is deposited in the Gene Expression Omnibus (GEO) (https://www.ncbi.nlm.nih.gov/geo/). GSE86278 has miRNA expression data measured in 46 Mexican patients with [miRNA-3] Affymetrix Multispecies miRNA-3 Array. These samples had the following classification: luminal A (*n* = 10), luminal B (*n* = 9), HER2 (*n* = 4), and triple negative (*n* = 23) (Figure S1). No other type of data, apart from miRNA expression and subtype classification, was available for this dataset.

### 4.2. Analysis of Differentially Expressed Genes

To find those DU-Exp genes between samples with and without Amp-650, we took advantage of the tools provided in the cBioportal website and performed Student’s *t*-test on more than 18,000 genes with mRNA expression data available. Since it was not possible to combine expression data from different transcriptomic platforms as a single unified dataset, we performed the analysis parallelly with both METABRIC and TCGA platforms. We repeated this process several times, each time making comparisons of different combinations of samples (Table 3).

To find potential miR-650 targeted TSG from the DU-Exp gene list, we first consulted the human Tumor Suppressor Gene Database and extracted a list of 983 candidates. According to this Database, these genes have either been reported as TSG and/or have a lower expression in the TCGA tumor samples than in the normal tissue samples (Table S4). We then compared all TSG between the DU-Exp gene list and conserved only TSG DU-Exp genes in samples with Amp-650. After gene filtration, every gene was assigned with the respective number of tools that had predicted them as miR-650 targets according to miRWalk 2.0 (See Target prediction and enrichment pathway analysis). We performed correlation matrix on DU-Exp putative target genes of miR-650 with R programming language (version 3.6.0) and statistically evaluated correlations with spearman method (*p*-value < 0.01).

For the GSE86278 dataset, we used the GEO2R tool available in GEO to perform differential expression analysis. In order to set a two-group comparison among the samples, we merged triple negative and HER2 since there were only four HER2 samples and both are aggressive subtypes, similarly, we merged luminal A and luminal B since both have heightened epithelial signature and are nonaggressive subtypes.

### 4.3. Evaluation of Statistical Difference between Proportions of Two Populations with Z-Test

To test statistical differences between different frequencies of amplification, we relied on the next equations:

(1) $$p=\frac{X_{1}+X_{2}}{n_{1}+\;n_{1}}$$

(2) $$\sigma_{p_{1}-p_{2}}=\sqrt{\frac{p\left(1-p\right)}{n_{1}}}+\frac{p\left(1-p\right)}{n_{2}}$$

(3) $$Z=\frac{(X_{1}/n_{1})-\left(X_{2}/n_{2}\right)}{\sigma_{p_{1}-p_{2}}}$$

In which *X*_1_ and *X*_2_, stand for the number of samples that harbor the Amp-650 within the first (*n*_1_) and second (*n*_2_) groups, respectively.

When choosing a confidence interval of 95% we have to consider α = 0.05 and 1 − α = 0.95. We looked for α = 0.05 and 1 − α = 0.95 in a conventional table for area under the one-tailed normal distribution curve *p* (Z ≤ Z0), and according to the table, the condition Z > 1.645 must be accomplished to consider that there is statistical difference between proportions from two different groups.

### 4.4. Target Prediction and Pathway Enrichment Analysis

We used miRWalk 2.0 (http://zmf.umm.uni-heidelberg.de/apps/zmf/mirwalk2/index.html) [47] to predict potential target genes for miR-650, a software that gathers 12 different “in silico” prediction tools. Each tool relies its prediction of targets based on any mRNA containing a complementary sequence within the 3′-UTR to the miRNA seed region. Prediction tools only differ on the weight assigned to every parameter taken into account for prediction. We obtained 17,857 different possible target genes predicted by at least one and up to 11 different tools (Table S2). All of the 17,857 possible target genes were considered for further analysis; however, we granted more importance to those predicted by at least six of the 12 prediction tools. String (https://string-db.org/) [48] was used to perform interaction networks and pathway enrichment analysis on the putative target genes of miR-650. Our setting specifications were as follows. Required interaction score: highest confidence (0.9), first shell: no more than 20 interactors, second shell: none; the rest of settings were left as default. After network construction STAT5A, THBD, and PER2 ended up with a combined score above 0.98. Table S3 shows the more informative and less redundant enriched pathways from Biological Process (GO) and KEGG with a false discovery rate less than 0.001, both available through String.

### 4.5. Survival Analysis

We performed survival analysis in R programming language (version 3.6.0) using the survival package and survminer package [49,50] to generate survival curve figures [51]. To determine samples overexpressing a gene of interest, a Z-score equal or more than 1 was set as threshold, while samples with a Z-score equal or less than -1 were considered to be underexpressing the gene of interest. These thresholds were used in all the three transcriptomic platforms available in cBioportal for BRCA.

### 4.6. PubMed Search

We searched in PubMed for frequently reported miRNAs as either amplified or overexpressed in BRCA samples, and we selected three microRNAs (miR-21, miR-210, and miR-10B) to use as reference to compare their frequencies of amplification to that of miR-650 within the BRCA population. We used the following key terms to perform our search:

[TI/AB] (breast OR mammary);
AND [TI/AB] (cancer OR carcinoma OR tumor);
AND [TI/AB] (miR OR miRNA OR microRNA);
AND [TI/AB] (overexpressed OR overexpression OR upregulated OR upregulation).

## Figures and Tables

**Table 1:** Summary of data available for each breast cancer study consulted in cBioportal.

Study	METABRIC [17]	TCGA (Firehose Legacy) [Not Published]	Metastatic Breast Cancer (INSERM) [18]	The Metastatic Breast Cancer Project (Provisional) [Not Published]
Data type	Number of samples			
miR-650 CNA	2173	1080	216	237
Molecular subtype	1980	522	N/A	N/A
TNM staging	1466	1086	N/A	110
Ethnicity	N/A	922	N/A	N/A
NHG	1892	N/A	N/A	101
NPI	1980	N/A	N/A	N/A
HER2 status (IHC)	1980	727	N/A	62
Overall survival	1980	1095	N/A	N/A
mRNA expression (Illumina Human v3 microarray)	1904	N/A	N/A	N/A
mRNA expression (Agilent microarray)	N/A	526	N/A	N/A
mRNA expression (RNA Seq V2 RSEM)	N/A	1100	N/A	N/A

N/A: not available. IHC: immunohistochemistry.

**Table 2:** Amplification/deletion of different miRNAs with a documented role in BRCA.

microRNA	Samples with Amplifications (*n* = 3706)	Samples with Deep Deletion (*n* = 3706)	PubMed Hits
miR-21	30.6%	0.1%	125
miR-210	9.3%	0.8%	23
**miR-650**	**7.1%**	**0.1%**	**1 (our group [12])**
miR-10B	6%	0%	39

**Table 3:** Samples available for each group used for the differential expression analysis.

	Study	METABRIC	TCGA (Firehose Legacy)	
	Transcriptomic platform	Illumina Human v3 microarray	Agilent microarray	RNA Seq V2 RSEM
Comparison name	Groups of samples	Number of samples		
Total Amp-650	Total samples with Amp-650	82	132	72
	Total samples w/o Amp-650	1822	961	454
BASAL	Basal with Amp-650	18	19	19
	Basal w/o Amp-650	181	78	76
HER2	HER2-enriched with Amp-650	18	18	18
	HER2-enriched w/o Amp-650	202	40	40
LumB	Luminal B with Amp-650	21	24	24
	Luminal B w/o Amp-650	440	103	103
LumA	Luminal A with Amp-650	14	9 *	9 *
	Luminal A w/o Amp-650	665	222	222
BASAL+HER2	Basal + HER2-enriched with Amp-650	36	37	37
	Basal + HER2-enriched w/o Amp-650	383	118	116
Lum B+A	Luminal B + Luminal A with Amp-650	35	33	33
	Luminal B + Luminal A w/o Amp-650	1105	325	325
CLAUDIN-LOW	Claudin-low with Amp-650	9 *	N/A	N/A
	Claudin-low w/o Amp-650	190	N/A	N/A
GRADE1	NHG 1 with Amp-650	2 *	N/A	N/A
	NHG 1 w/o Amp-650	163	N/A	N/A
GRADE2	NHG 2 with Amp-650	20	N/A	N/A
	NHG 2 w/o Amp-650	720	N/A	N/A
GRADE3	NHG 3 with Amp-650	56	N/A	N/A
	NHG 3 w/o Amp-650	869	N/A	N/A

*Numbers excluded from the analysis because were considered too small to obtain reliable Student’s *t*-test results. N/A: not available. Total Amp-650 groups were made regardless of any clinical classification.

**Table 4:** Summary of DU-Exp genes and the BRCA subtypes in which they are underexpressed.

Comparison	BASAL	BASAL+ HER2	GRADE3	GRADE2	Number of Predicting Tools (Total = 12)
STAT5A	3.35 × 10^−3^	2.23 × 10^−3^	1.61 × 10^−4^	ns	6
THBD	4.04 × 10^−3^	ns	ns	8.56 × 10^−4^	7
PER2	ns	ns	1.86 × 10^−3^	ns	8

Basal and HER2 subtypes were compared against Luminal; Grade 3 against Grade 2. Only *p*-values < 0.01 are presented. ns: not significant.

## Supplementary Materials

Supplementary Materials can be found at https://www.mdpi.com/1422-0067/21/20/7720/s1.

## Abbreviations

BRCA	breast cancer
EMT	epithelial to mesenchymal transition
HER2	human epidermal growth factor 2
NHG	Neoplasm Histologic Grade
NPI	Nottingham Prognostic Index
miRNAs	microRNAs
TSG	tumor suppressor genes
Amp-650	amplification of miR-650
DU-Exp	differentially underexpressed
CNA	copy number alterations
OS	overall survival
