# Supplementary material for: A Bioinformatic Pipeline Places STAT5A as a miR-650 Target in Poorly Differentiated Aggressive Breast Cancer
Source: Int J Mol Sci. 2020 Oct 19;21(20):7720. doi: 10.3390/ijms21207720 (PMC7589888; doi:10.3390/ijms21207720)
Supplement: Supplementary file 1 [file ijms-21-07720-s001.zip › ijms-963600-supplementary/ijms-963600-Figure S.pdf]

A bioinformatic pipeline places STAT5A as a miR-650 target in poorly differentiated aggressive breast cancer

Eric López-Huerta,[1,2] and Ezequiel M. Fuentes-Pananá[2,*]

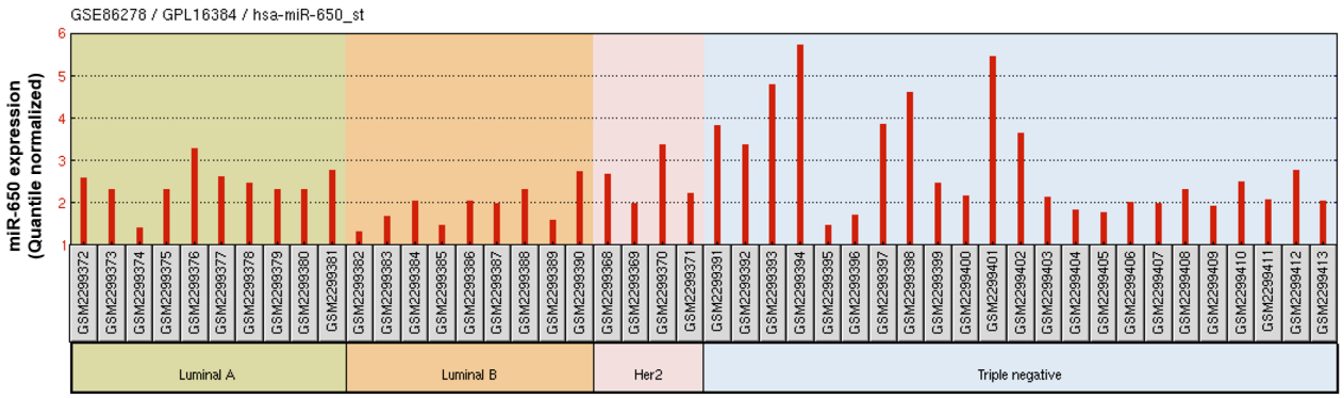

Figure S1: Expression levels of miR-650 among 46 Mexican patient samples from the GSE86278 dataset. Plot generated with "Profile graph" option of GEO2R available in GEO.

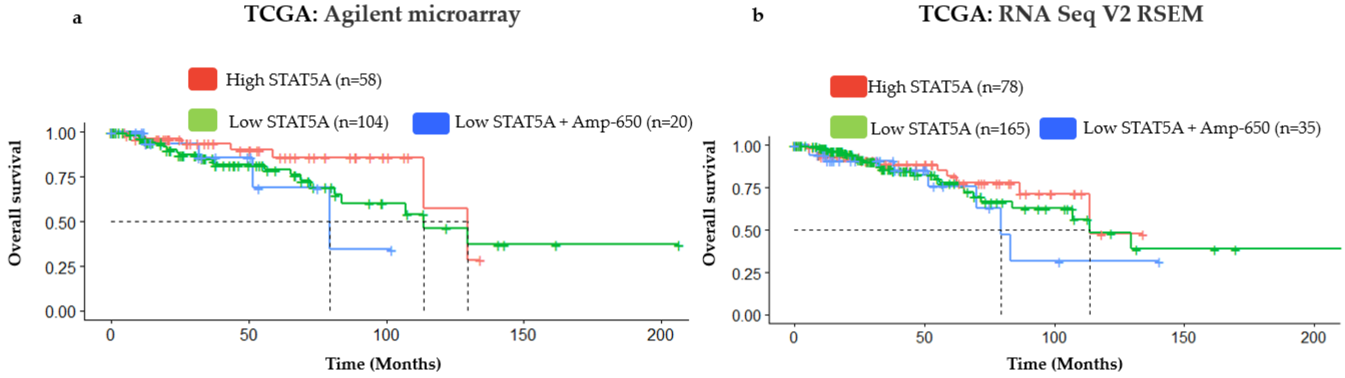

Figure S2: Prognostic significance of Amp-650 in combination with the expression level of STAT5A from TCGA data. Curves were constructed using: (a) Agilent microarray and (b) RNA Seq V2 RSEM, both from TCGA. Z-score was used to set the level of STAT5A expression as in figure 4. Group of High STAT5A + Amp-650 was not considered because of the small number of samples (n=9 for Agilent microarray and n=8 for RNA Seq V2 RSEM data). Dashed lines indicate median OS. No statistical differences were found between any group.

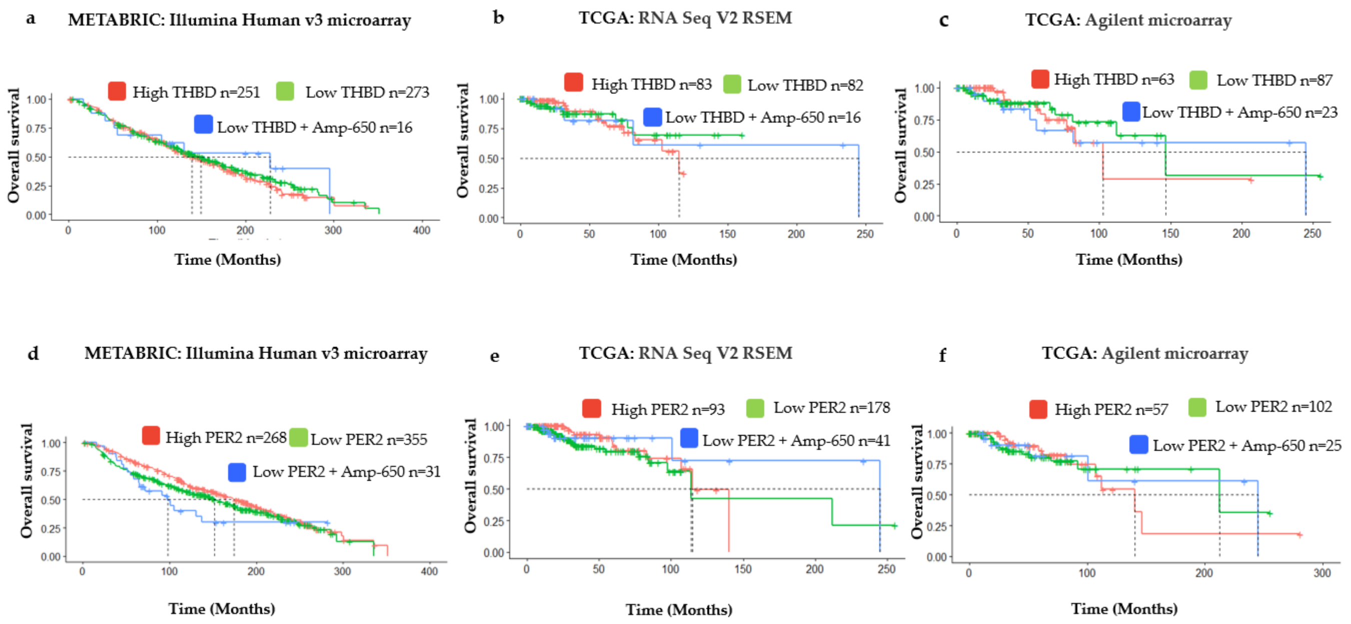

Figure S3: Prognostic significance of Amp-650 in combination with the expression level of THBD (a-c) and PER2 (d-f). Above each curve is indicated the expression data used in its construction. Z-score was used to set high expressing samples (above +1) or low expressing samples (below -1) of THBD/PER2. High gene expression and Amp-650 comparisons were not done because the small number of samples. Dashed lines indicate median OS. No statistical differences were found between any group.

Table S1: Summary of target genes of miR-650 in solid cancers and the number of tools that predicted them as targets.

miR-650 target gene	Number of predicting tools	References
LATS2	7	[30]
ING4	7	[13], [15]
NDRG2	6	[14]
SCARA3	6	[16]
PPP2CA	3	[29]

Table S2: Summary of results obtained with miRWalk2.0

Number of prediction tools	Sum of target genes
12	0
11	5
10	41
9	164
8	550
7	1130
6	1360
5	1643
4	2030
3	2874
2	4309
1	3751
